# A preliminary analysis of interleukin-1 ligands as potential predictive biomarkers of response to cetuximab

**DOI:** 10.1186/s40364-019-0164-0

**Published:** 2019-07-16

**Authors:** Madelyn Espinosa-Cotton, Elana J. Fertig, Laura P. Stabile, Autumn Gaither-Davis, Julie E. Bauman, Sandra Schmitz, Katherine N. Gibson-Corley, Yinwen Cheng, Isaac J. Jensen, Vladimir P. Badovinac, Douglas Laux, Andrean L. Simons

**Affiliations:** 1 Free Radical and Radiation Biology Program, Department of Radiation Oncology, University of Iowa, Iowa City, IA USA; 2 Department of Pathology, 1161 Medical Laboratories, University of Iowa, Iowa City, IA 52242 USA; 3 Holden Comprehensive Cancer Center, University of Iowa Hospitals and Clinics, Iowa City, IA USA; 4 Division of Biostatistics and Bioinformatics, Department of Oncology, Sidney Kimmel Comprehensive Cancer Center, Johns Hopkins University, Baltimore, MD USA; 5 Department of Pharmacology and Chemical Biology, University of Pittsburgh, Pittsburgh, PA USA; 6 Division of Hematology and Oncology, University of Arizona Cancer Center, Tucson, AZ USA; 7 Université catholique de Louvain, Louvain-la-Neuve, Belgium; 8 Interdisciplinary Graduate Program in Human Toxicology, University of Iowa, Iowa City, IA USA; 9 Interdisciplinary Immunology Graduate Program, University of Iowa, Iowa City, IA USA; 10 Department of Microbiology and Immunology, University of Iowa, Iowa City, IA USA; 11 Department of Internal Medicine - Hematology, Oncology and Blood and Marrow Transplantation, University of Iowa Hospitals and Clinics, Iowa City, IA USA

**Keywords:** Cetuximab, Interleukin-1 (IL-1), Head and neck squamous cell carcinoma (HNSCC), Biomarker

## Abstract

**Background:**

The epidermal growth factor receptor (EGFR) monoclonal IgG_1_ antibody cetuximab is approved for first-line treatment of recurrent and metastatic (R/M) HNSCC as a part of the standard of care EXTREME regimen (platinum/5-fluorouracil/cetuximab). This regimen has relatively high response and disease control rates but is generally not curative and many patients will experience recurrent disease and/or metastasis. Therefore, there is a great need to identify predictive biomarkers for recurrence and disease progression in cetuximab-treated HNSCC patients to facilitate patient management and allow for treatment modification. The goal of this work is to assess the potential of activating interleukin-1 (IL-1) ligands (IL-1 alpha [IL-1α], IL-1 beta [IL-1β]) as predictive biomarkers of survival outcomes in HNSCC patients treated with cetuximab-based chemotherapy.

**Methods:**

Baseline gene, serum and tumor expression of interleukin-1 (IL-1) ligands were analyzed from The Cancer Genome Atlas (TCGA) database or clinical trials of cetuximab-based therapies and interrogated for associations with clinical outcome data.

**Results:**

High tumor gene expression of IL-1β was associated with a more favorable overall survival in cetuximab-treated HNSCC patients but not in non-cetuximab-treated patients. In HNSCC patients treated with cetuximab-based chemotherapy, higher gene and circulating levels of IL-1α and IL-1β were correlated with a more favorable progression free survival compared to patients with low or undetectable levels of IL-1 ligands.

**Conclusions:**

These findings suggest that IL-1 ligands may function as predictive biomarkers for tumor response to cetuximab-based chemotherapy in HNSCC patients and warrants further investigation and validation in larger clinical studies.

## Background

Cetuximab is a human/mouse chimeric antibody on an IgG_1_ backbone that inhibits EGFR signaling by interfering with the binding of EGFR ligands (EGF, TGFα) to EGFR and also by the depletion of EGFR from the cell membrane via receptor endocytosis [1]. In addition to cetuximab’s effects on EGFR, its IgG_1_ backbone triggers additional antitumor activity in the form of natural killer (NK) cell-mediated antibody-dependent cell-mediated cytotoxicity (ADCC) [2–4]. Cetuximab-activated NK cells are also able to promote dendritic cell (DC) maturation and CD8+ T-cell activation [5] suggesting that cetuximab can promote anti-tumor immunity in HNSCC patients. Cetuximab is FDA approved for HNSCC treatment based on its ability to increase survival in combination with radiation for locally advanced disease [6] and improve response rates in combination with chemotherapy (cisplatin ±5-FU) for R/M disease [7].

To date, there are no biomarkers used in clinical practice that can predict tumor response to cetuximab in HNSCC patients. Predictive biomarkers of tumor response to EGFR inhibitors have been well established in non-small cell lung cancer (NSCLC) [8] and colorectal cancer (CRC) [9] but these mechanisms (including EGFR and KRAS mutations) are extremely rare and are not clinically relevant to HNSCC [10, 11]. Numerous preclinical studies have identified potential predictive biomarkers of EGFR inhibitor response in HNSCC including alterations in EGFR ligand expression levels [12–14], EGFR polymorphisms [15], EGFR variant III expression [16], nuclear EGFR [17], overexpression of other ErbB family receptors [18], MET activation [19], Axl overexpression [20], alterations in targets downstream of EGFR (e.g. PI3K, PTEN, src) [21] and FcγR SNPs [22]. However, efforts to validate or target these proposed mechanisms in clinical trials have yielded modest results or have failed. Recent work has identified a germline, functional single-nucleotide polymorphism in the KRAS 3′-untranslated region (rs61764370) in 17% of HNSCC patients which leads to gain of function phenotype [23]. Interestingly, unlike activating KRAS mutations in CRC, the presence of this “KRAS-variant” was not found to be associated with resistance to cetuximab but rather favorable outcomes [23]. While these results are promising, it does not explain differences in tumor response to cetuximab in the majority of HNSCC patients lacking the KRAS variant. Altogether, it is clear that further investigation is warranted to further identify strategies that will predict and enhance tumor response to cetuximab for R/M HNSCC patients given its importance in standard of care.

Our previous work indicated that EGFR inhibition triggers activation of the interleukin-1 (IL-1) pathway [24, 25] although the clinical implications of this pathway are unclear. The IL-1 pathway plays a central role in immune and inflammatory responses by regulating the expression of various inflammatory genes in immune cells. This pathway is triggered when the ligands IL-1α and IL-1β bind to the IL-1 receptor type 1 (IL-1R1) [26]. Upon ligand binding, the receptor forms a complex with the IL-1 receptor accessory protein, leading to the recruitment of MyD88, IL-1 receptor-associated kinases and TRAF6 [26]. These signaling events prompt NFkB and MAPK signaling leading to the expression of IL-1 target genes.

IL-1 signaling has been reported in various studies to be associated with poor prognosis due to the resulting downstream expression of genes involved in tumor progression [25, 27–31]. In contradiction to IL-1’s tumor-promoting role, IL-1 signaling has been shown to be involved in tumor cell killing via an anti-tumor immune response [32–34]. IL-1 signaling is proposed as a key mediator of host defense against malignancies mainly through its role on NK cell activity (i.e. IFNγ production and ADCC) [32] and NK-cell activity can be significantly inhibited by anakinra (IL-1RA), or by neutralizing antibodies for IL-1 ligands [35]. IL-1 was shown to directly enhance survival of CD4+ T cells and induce secondary CD8+ T cell responses characterized by enhanced granzyme B expression and increased IFNγ production [36–38].

Given that cetuximab’s anti-tumor activity is in part due to natural killer (NK) and T cell-mediated cytotoxicity [1, 3, 39, 40] and IL-1 signaling can activate NK and T cell anti-tumor immune responses [41–44], we propose that increased tumor and/or circulating levels of IL-1 ligands would be indicative of a strong anti-tumor immune response leading to favorable and perhaps durable responses to chemotherapy regimens that involve cetuximab. Here we show preliminary evidence in limited cohorts of HNSCC patients that higher gene and circulating levels of IL-1α and IL-1β are correlated with favorable progression free survival in HNSCC patients treated with cetuximab-based chemotherapy compared to patients with low or undetectable levels of IL-1 ligands. These findings suggest that IL-1 ligands may be promising as predictive biomarkers for survival outcomes in cetuximab-based chemotherapy-treated HNSCC patients.

## Methods

### Analysis of TCGA dataset

The Cancer Genome Browser (University of California – Santa Cruz) was used to download a level 3 head and neck cancer dataset (TCGA_HNSC_exp_HiSeqV2–2015-02-24) from the Cancer Genome Atlas, along with corresponding clinical data including treatment history. Only HNSCC patients (*n* = 164) in the database where treatment information was included were included in these analyses. These patients were then separated into patients that included cetuximab in their therapy regiment (*n* = 34) and patients that did not include cetuximab in their therapy regimen (*n* = 130) and ranked according to gene expression for each gene of interest in their tumors, with the upper half denoted as “high *gene x*” and the lower half as “low *gene x”*. None of these tumors were RAS mutants. Kaplan-Meier survival curves were plotted comparing the overall 3-year survival of patients with high or low tumor expression of various genes. A subset of the patients in the dataset (*n* = 42) also had gene expression analysis data from adjacent normal tissue. For these patients, expression of IL-1 ligands was plotted for matched tumor vs. normal tissue.

### Patient tumor IL-1 gene expression analysis

Publically available global gene expression data from an Illumina HumanHT-12_v4 expression beadchip array for R/M HNSCC patients treated with first-line cetuximab+chemotherapy (i.e. cisplatin, cisplatin+ 5-FU, cisplatin+paclitaxel, cisplatin+pemetrexed) [45] was analyzed. As described [45], patients with greater than 12 month progression free survival (PFS) were designated as those with long PFS and patients with shorter than 5.6 months PFS were designated as those with short PFS. Log transformed, pre-processed gene expression values in the series matrix from GEO (GSE65021) were used for analysis. Differential expression analysis was performed between patients with long and short PFS using LIMMA version 3.30.13 [45]. In this analysis, a single probe was selected for each gene with the R/Bioconductor annotation package IlluminaHumanv4.db version 1.26.0. Analysis was performed on IL-1 ligands ((*IL1A*, ILMN_1658483; *IL1B*, ILMN_775501) and reported *p*-values are false discovery rate (FDR)-adjusted with Benjamini-Hotchberg on unadjusted p-valued for this set of ligands.

### Availability of data and material

Datasets from the TCGA (TCGA_HNSC_exp_HiSeqV2–2015-02-24) and GEO databases (http://www.ncbi.nlm.nih.gov/geo/, accession number GSE65021) are publically available. The datasets generated during and/or analyzed during the current study are available from the relevant authors on reasonable request.

### Analysis of R/M HNSCC patient serum samples

Baseline serum samples from R/M HNSCC patients scheduled for cetuximab-based chemotherapy (i.e. carboplatin, cisplatin, 5-FU, paclitaxel) at the University of Iowa Hospitals and Clinics (UIHC) Holden Comprehensive Cancer Center and R/M cetuximab-resistant HNSCC patients scheduled for cetuximab in combination with ficlatuzumab (human growth factor [HGF] antibody) in a phase I study (ClinicalTrials.gov Identifier: NCT03422536) were collected. Serum IL-1 ligand (IL-1α, IL-1β) levels were measured by enzyme-linked immunosorbent assays (ELISAs). Both studies were approved by the respective institution’s Institutional Review Board and was conducted in accordance with ethical standards presented in the 2013 Declaration of Helsinki. All subjects provided their informed consent in written form for participation in the study.

### ELISAs on human serum/plasma

DuoSet ELISAs for IL-1α and IL-1β were purchased from R&D Systems (Minneapolis, MN, USA) and carried out according to manufacturer’s protocols. The lower limits for detection were as follows: IL-1α – 7.8 pg/mL and IL-1β - 3.9 pg/mL.

### Flow cytometry

Normal human peripheral blood mononuclear cells (PBMCs) were cultured at 10:1 and 1:1 effector/target ratio for 6 h at 37 °C with SQ20B cells that were treated with cetuximab with or without anakinra. Culture media containing PBMCs were then stained with anti-CD3, anti-CD16, anti-CD54 and anti-CD107a antibodies conjugated to different fluorochromes. NK cells, gated on CD3− cells in the lymphocyte FSC/SSC subset were analyzed for the expression of CD16 (A) and CD54 (B) and CD107a (C). Cells were collected on a FACSCanto flow cytometer. All data were analyzed using FlowJo software.

### Tumor cell implantation

Female athymic *nu/nu* (4–6 weeks old) were purchased from Envigo Laboratories (Huntingdon, Cambridgeshire, United Kingdom). Mice were housed in a pathogen-free barrier room in the Animal Care Facility at the University of Iowa and handled using aseptic procedures. Mice were allowed at least 3 days to acclimate prior to beginning experimentation, and food and water were made freely available. SQ20B (1 × 10^6^ cells/mouse) were inoculated into athymic nude mice by subcutaneous injection of 0.1 mL aliquots of saline containing cancer cells into the right flank using 26 gauge needles.

### In vivo drug administration

Drug treatment commenced 3 days after tumor inoculation. Female SQ20B tumor-bearing athymic *nu/nu* mice (*n* = 6 mice/treatment group) were randomized and treated either an IL-1R antagonist (anakinra [ANA]) at 10 mg/kg i.p. daily, cetuximab (CTX) at 2 mg/kg i.p. twice per week, or CTX + ANA i.p. at the doses/schedules indicated above. Mice were administered saline daily and 2 mg/kg IgG i.p twice per week as a control. All treatments were given for 3 weeks. Mice were evaluated daily and tumor measurements and weights were taken three times per week using Vernier calipers. Tumor volumes were calculated using the formula: tumor volume = (length × width^2^)/2. Mice were euthanized via CO_2_ gas asphyxiation when tumor diameter exceeded 1.5 cm in any dimension. Tumor growth curves were plotted over time and stopped after a mouse in any treatment group reached euthanasia criteria.

### Statistical analysis

Statistical analysis was carried out using GraphPad Prism version 7 for Mac (GraphPad Software, La Jolla, CA, USA). Differences in means between two groups were determined by unpaired t-test. Kaplan-Meier survival curves were generated to illustrate the different survival rates over time. Differences in survival were determined by Log-rank (Mantel-Cox) test. For all experiments, differences were considered significant if *p* < 0.05.

## Results

### Tumor gene expression of IL-1 ligands may positively predict survival in patients treated with cetuximab

**Fig. 1:**
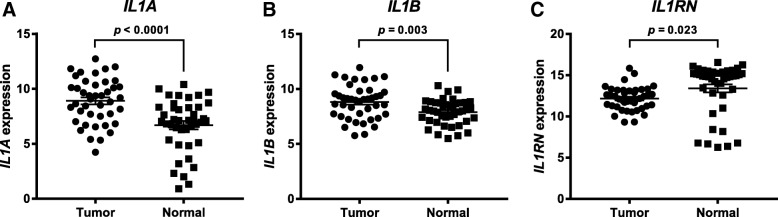
Expression of IL-1 ligands is elevated in head and neck tumor versus normal tissue. Gene expression of *IL1A* (**a**), *IL1B* (**b**), and *IL1RN* (**c**) from tumor (*n* = 42) and matched adjacent normal tissue was plotted for available HNSCC patients from the TCGA database

**Fig. 2:**
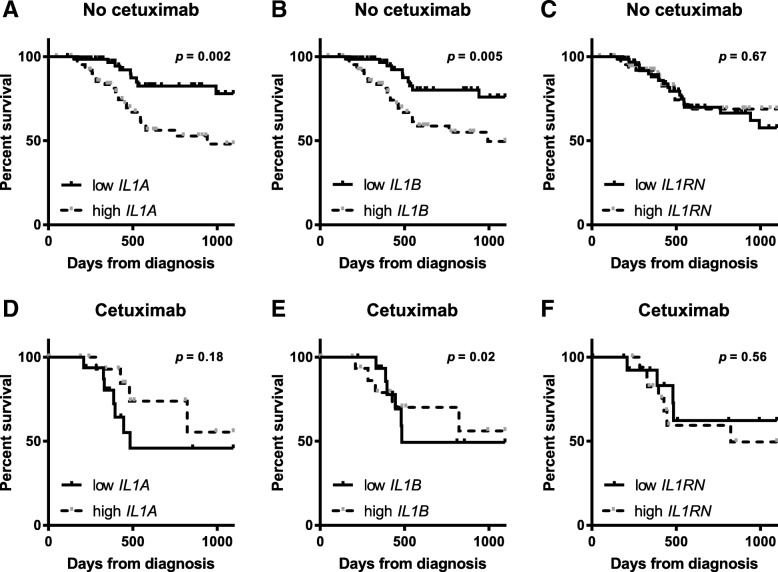
Tumor gene expression of IL-1 ligands may positively predict survival in patients treated with cetuximab. Shown are Kaplan-Meier survival curves comparing overall survival of HNSCC patients treated with “non-cetuximab based therapy” (No cetuximab) (n = 130 [**a**-**c**]) according to with high (*n* = 64) or low (*n* = 66) *IL1A* (**a**), *IL1B* (**b**) and *IL1RN* (**c**) tumor gene expression; and HNSCC patients treated with “cetuximab based therapy” (Cetuximab) (n = 34 [**d**-**f**]) according to with high (*n* = 18) or low (*n* = 16) *IL1A* (**a**), *IL1B* (**b**) and *IL1RN* (**c**) tumor gene expression

RNA-sequencing data for HNSCC tumors and matched normal tissue (*n* = 42) were analyzed from the TCGA for mRNA levels of the IL-1 ligands IL-1α, IL-1β and IL-1RA. *IL1A* and *IL1B* were found to be significantly increased in tumors (Fig. 1a,b *p* < 0.0001 and *p* = 0.003, respectively) compared to normal samples while *IL1RN* (IL-1RA) was significantly decreased in tumor versus normal tumors (Fig. 1c, *p* = 0.02). RNA-sequencing data for HNSCC tumors (*n* = 164) from the TCGA (with associated and complete clinical outcome data) with high expression of IL-1 ligands were plotted for survival against low IL-1 ligand expressing tumors. Gene expression analyses were performed on tumor specimens harvested before treatment with chemotherapy or radiation. In patients whose therapy did not include cetuximab (*n* = 34), high gene levels of *IL1A* and *IL1B* were significantly associated with worse survival compared to low gene levels (Fig. 2a,b, *p* = 0.002 and 0.005, respectively). On the other hand, in patients who were treated with cetuximab-based therapy (*n* = 130), high *IL1A* and *IL1B* gene levels were not associated with worse survival (Fig. 2d,e, *p* = 0.18 and 0.02, respectively). In fact, *IL1A* and *IL1B* appeared to be associated with a more favorable survival in cetuximab-based therapy-treated patients although only the differences in *IL1B* reached significance (Fig. 2e, *p* = 0.02). There was no difference in survival outcomes for cetuximab- or non-cetuximab-treated patients on the basis of *IL1RN* tumor expression (Fig. 2c,f, *p* = 0.67 and 0.55, respectively). Analysis of corresponding patient characteristics and available clinicopathological data indicated no differences between the cetuximab-based therapy versus non-cetuximab-based therapy-treated patient cohorts on the basis of sex, age, HPV status, treatment with radiotherapy, or proportion of early/late stage cases (Table 1). However, of the HNSCC patients analyzed, cetuximab-based therapy was associated with a significantly higher likelihood of relapse and a less complete treatment responses compared to non-cetuximab-based therapy (Table 1). Overall, these findings prompted the further evaluation of *IL1A* and *IL1B* gene expression and their association with survival outcomes in cetuximab-based therapy-treated HNSCC patients.

**Table 1:** HNSCC Patient Characteristics

Patient characteristics	cetuximab (*n* = 34) % (n)	no cetuximab (*n* = 130) % (n)	*p* value
Male	71% (24)	83% (108)	*p* = 0.10
Female	29% (10)	17% (22)	
Mean age [range]	58.8 [35–82]	56.3 [19–80]	*p* = 0.22
Stage at diagnosis			
I, II	12% (4)	9% (12)	*p* = 0.67
III, IV	88% (30)	90% (117)	
Unknown	0% (0)	1% (1)	
HPV status			
Positive	15% (5)	18% (24)	*p* = 0.85
Negative	26% (9)	26% (34)	
Unknown	59% (20)	52% (70)	
Relapsed			
Yes	53% (18)	31% (40)	*p* = 0.043
No	35% (12)	58% (75)	
Unknown	12% (4)	12% (15)	
Received radiation therapy			
Yes	74% (25)	77% (100)	*p* = 0.92
No	12% (4)	10% (13)	
Unknown	15% (5)	13% (17)	
Treatment best response			
Complete response	26% (9)	42% (55)	*p* = 0.004
Partial response	3% (1)	2% (3)	
Stable disease	3% (1)	1% (1)	
Progressive disease	21% (7)	3% (4)	
Unknown	47% (16)	53% (69)	

### Tumor gene expression of IL-1 ligands predict progression-free survival (PFS) in patients treated with cetuximab and chemotherapy

**Fig. 3:**
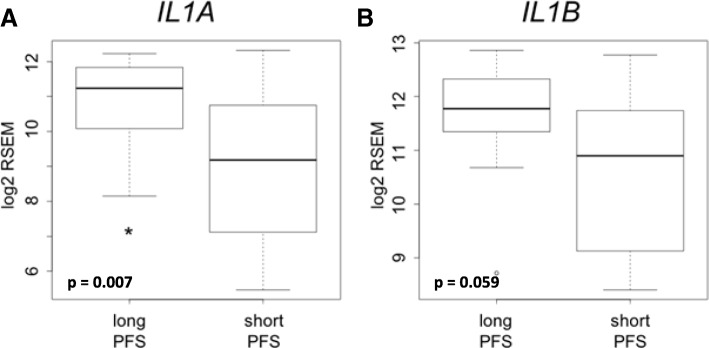
Tumor gene expression of IL-1 ligands predicts PFS in patients treated with cetuximab and chemotherapy. Comparison of pre-treatment tumor gene expression of *IL1A* (**a**) and *IL1B* (**b**) in HNSCC patients treated with first-line cetuximab-based chemotherapy separated into long progression free survival (PFS) (> 12 months, *n* = 14) and short PFS (< 5.6 months, *n* = 26)

Gene expression of IL-1 ligands were retrospectively determined from pre-treatment tumor biopsies from 40 R/M HNSCC patients who were treated with first-line cetuximab-based chemotherapy [46]. In this published study the authors separated the patients into those with long PFS (> 12 months, median = 19 months, *n* = 14) and short PFS (< 5.6 months, median = 3 months, *n* = 26) [46]. *IL1A* (Fig. 3a, FDR-adjusted *p* = 0.007) was significantly higher in patients with long PFS compared to short PFS, while *IL1B* trended higher in patients with long PFS but fell short of statistical significance (Fig. 3b, FDR-adjusted *p* = 0.059). There were no differences in demographic or other clinicopathological parameters in long PFS vs short PFS patients as already previously reported [46]. Together, the results shown in Figs. 2 and 3 suggest a possible connection between IL-1 ligand expression and response to cetuximab-based therapy.

### High serum IL-1 predicts progression-free survival in HNSCC patients treated with cetuximab-containing therapy

**Fig. 4:**
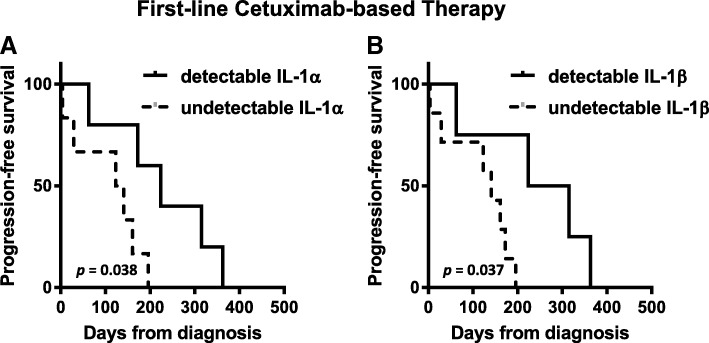
High serum IL-1 may predict favorable PFS in HNSCC patients treated with cetuximab-containing therapy. Baseline serum samples from R/M HNSCC patients scheduled for cetuximab-based chemotherapy were collected and serum levels of IL-1α (**a**) and IL-1β (**b**) levels were measured by ELISA. Progression free survival was compared for patients with “detectable” versus “undetectable” serum levels of IL-1α (*n* = 5 vs n = 6 respectively) and IL-1β (n = 4 vs *n* = 7 respectively)

To determine if circulating IL-1 ligand levels would predict survival outcomes in cetuximab-based chemotherapy-treated HNSCC patients we analyzed pre-treatment serum samples from 11 consented patients who were treated with cetuximab-based chemotherapy (i.e. carboplatin, cisplatin, 5-FU, paclitaxel) and have available and complete clinical outcome data. IL-1 ligand levels varied widely among the patients and ranged from undetectable (0 pg/mL / below limit of detection) to 418 pg/mL (IL-1α) and 262 pg/mL (IL-1β). Six out of the 11 patients had undetectable baseline levels of IL-1 ligands. Differences between pre-treatment IL-1 ligand levels in patients with stable disease (SD, *n* = 6) compared to progressive disease (PD, *n* = 5) according to RECIST criteria did not meet significance (data not shown). None of the patients achieved a response of partial response (PR) or complete response (CR). However, when PFS data was analyzed there were significantly longer PFS times in patients with detectable levels of IL-1α (Fig. 4a, *p* = 0.038, n = 5) and IL-1β (Fig. 4b, *p* = 0.037, *n* = 4) compared to low IL-1 ligand levels using ELISA. These data suggest that circulating IL-1 ligand levels may be promising as a predictive indicator of PFS rather than short-term treatment response.

### High serum IL-1 levels may predict survival outcomes in cetuximab-resistant R/M HNSCC patients treated with cetuximab+ficlatuzumab

**Fig. 5:**
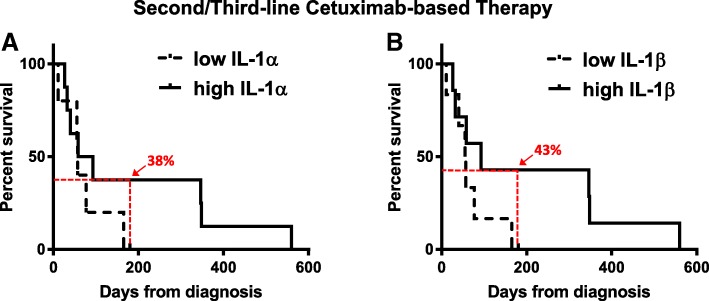
High serum IL-1 levels may predict survival outcomes in a subset of cetuximab-resistant R/M HNSCC patients treated with cetuximab+ficlatuzumab. IL-1α (**a**) and IL-1β (**b**) levels were measured by ELISA in baseline serum samples from R/M cetuximab-resistant HNSCC patients scheduled for cetuximab in combination with ficlatuzumab. Progression free survival was compared for patients with “high” versus “low” serum levels of IL-1α (*n* = 8 vs n = 5 respectively) and IL-1β (n = 7 vs n = 6 respectively)

We next determined if IL-1 ligand levels were associated with survival outcomes in R/M HNSCC patients that had previously progressed on cetuximab-based therapy and were scheduled to receive cetuximab in combination with the HGF monoclonal antibody ficlatuzumab [47] in a phase I clinical trial (NCT02277197). Two patients achieved PR, 6 achieved SD and 4 experienced PD. No significant differences were observed in IL-1 ligand levels between patients with SD, PD or PR (Fig. 5a,b). There was also no significant difference in the survival curves for the IL-1 ligands when analyzed by log-rank test (Fig. 5c,d, *p* = 0.27 and 0.128 respectively). However, a late divergence in the survival curves was observed for both IL-1α (6 month survival = 38% [high] vs. 0% [low]) and IL-1β (6 month survival = 43% [high] vs. 0% [low]) which was associated with a delayed therapy benefit in patients with high circulating IL-1 ligand levels (Fig. 5c,d). These data suggest that circulating IL-1 ligand levels may be a predictive indicator of PFS in a subset of cetuximab-refractory patients.

### IL-1 blockade may reduce the anti-tumor efficacy of cetuximab

**Fig. 6:**
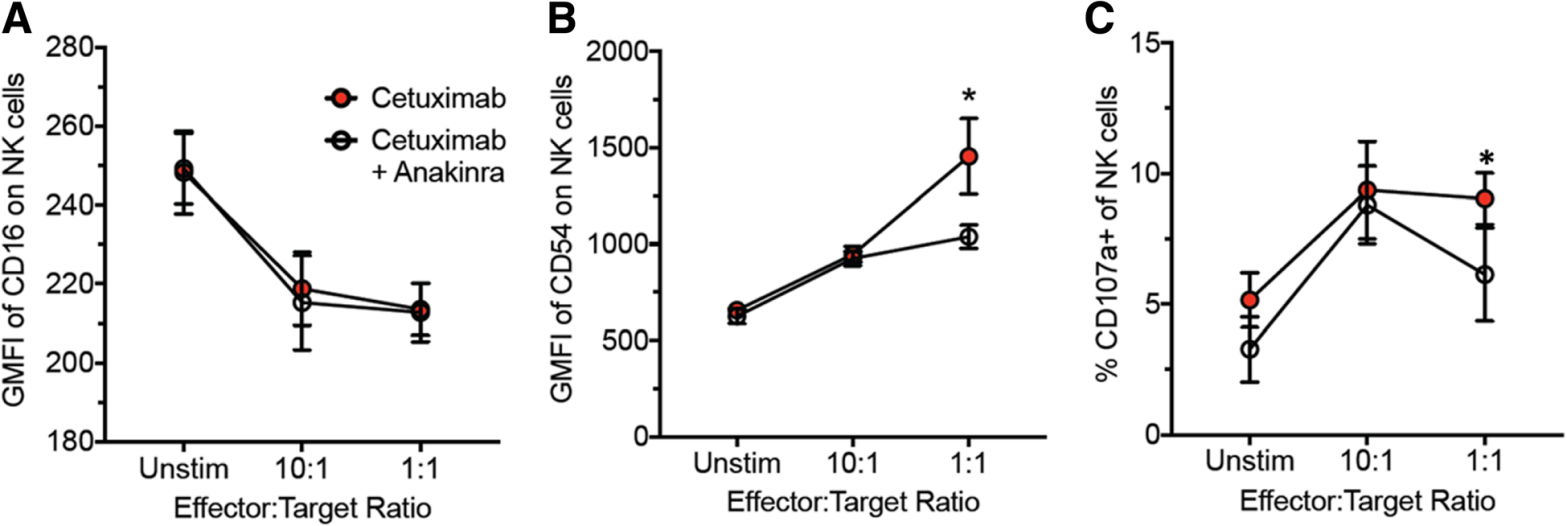
IL-1 blockade suppresses cetuximab-induced NK cell activity. Normal human peripheral blood mononuclear cells (PBMCs) were cultured at 10:1 and 1:1 effector/target ratio for 6 h at 37 °C with SQ20B cells that were treated with cetuximab with or without anakinra. Culture media containing PBMCs were then stained with anti-CD3, anti-CD16, anti-CD54 and anti-CD107a antibodies conjugated to different fluorochromes. NK cells, gated on CD3− cells in the lymphocyte FSC/SSC subset were analyzed for the expression of CD16 (**a**) and CD54 (**b**) and CD107a (**c**). Error bars = SD, *:*p* < 0.05 vs cetuximab+anakinra

**Fig. 7:**
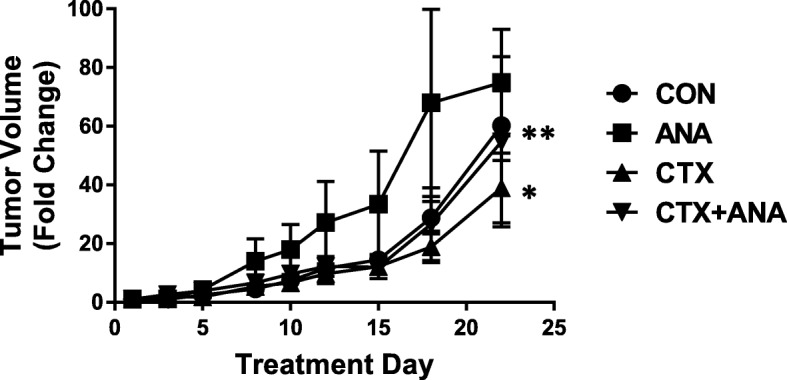
Role of IL-1 pathway blockade on the anti-tumor efficacy of cetuximab. Female athymic (*nu/nu*) mice bearing SQ20B xenograft tumors were treated with 2 mg/mouse cetuximab (CTX) twice/week i.p. with or without anakinra (IL-1RA) administered at 10 mg/kg daily i.p. for 3 weeks. IgG and PBS were used as controls. *N* = 6 mice/treatment group. Error bars represent ± SEM. *p < 0.05 versus control; **p < 0.05 versus CTX

In an effort to understand why PFS would be unfavorable in cetuximab-based therapy-treated HNSCC patients with low circulating IL-1 levels, we assessed NK cell activity identified by CD3-CD56 + CD54+ cells (activated NK cells) and CD3-CD53 + CD107a + (NK cell degranulation) by flow cytometry after co-culture of PBMCs with cetuximab and cetuximab+anakinra-treated SQ20B cells. We showed that IL-1 blockade using anakinra did not affect cetuximab binding to the Fc gamma-receptor III (FcγRIII, CD16) on NK cells (Fig. 6a) however, anakinra suppressed NK cell activation induced by cetuximab (Fig. 6b) and NK cell degranulation (Fig. 6c) when cultured at a 1:1 effector to target ratio. These results suggest that IL-1 signaling may be important for cetuximab-induced NK cell activity. Lastly, we showed in SQ20B tumor-bearing athymic nude mouse that anakinra (which binds both human and mouse ligands) partially reversed the anti-tumor effect of cetuximab treatment (Fig. 7). These results suggest that low/reduced levels of IL-1 ligands may suppress the ability of cetuximab to activate NK cell activity and thus reduce the anti-tumor efficacy of this agent.

Altogether, our results indicate that higher tumor gene expression and/or circulating levels of IL-1 ligands may be associated with favorable survival outcomes in cetuximab-based therapy-treated HNSCC patients. IL-1 ligands may serve as promising predictive biomarkers for long-term response to cetuximab-based therapy and warrants further validation in much larger patient cohorts.

## Discussion

The data from the limited cohorts presented here supports the idea of IL-1 ligands as predictive biomarkers of favorable clinical outcomes specifically in cetuximab-treated R/M HNSCC patients. IL-1 signaling is typically associated with an aggressive/malignant phenotype, poor prognosis and drug resistance in general [46, 48, 49]. Additionally, published reports from the CANTOS trial suggested that targeting IL-1β could reduce the incidence of lung cancer and lung cancer mortality [50]. Our survival analyses from the TCGA database showing high IL-1 ligand expression being associated with worse survival in non-cetuximab-treated HNSCC patients (Fig. 2a,b) supports the role of IL-1 in poor prognosis. However our findings of activating IL-1 ligands being associated with more favorable survival outcomes in cetuximab-treated HNSCC patients (Fig. 2d,e) challenge the current understanding of the pro-survival role of IL-1 signaling in tumor biology. The data set analyzed represents only the HNSCC patients from the TCGA database that were accompanied by information about their therapy regimen (Fig. 2). Therefore it is possible that by selecting only these patients our dataset may be biased. However the finding in this data set of a significantly higher like-hood of relapse and a less complete treatment response in cetuximab-treated HNSCC patients compared to non-cetuximab-treated patients supports results from a recent phase 3 trial (RTOG 1016) showing higher likelihood of locoregional failure and significantly lower overall survival and progression free survival in cetuximab-treated HNSCC patients compared to the non-cetuximab treated patients. This suggests that our data set may not be biased and perhaps may reflect realistic outcomes of cetuximab therapy. IL-1 signaling has the capability of activating both NK and T cell anti-tumor immune responses [33–35]. It is possible that this anti-tumor immunity role of IL-1 is beneficial for optimal anti-tumor efficacy of cetuximab since NK cell-mediated ADCC and CD8+ T cell activation are arguably the major mechanisms of action of cetuximab [2, 3, 5]. Increased circulating levels of activating IL-1 ligands would thus be indicative of a strong anti-tumor immune response which would be predictive of a favorable and perhaps durable response to cetuximab-based therapy.

Cetuximab is approved for first-line treatment of R/M HNSCC as a part of the standard of care EXTREME regimen (cetuximab+platinum±5-fluorouracil) [51]. This regimen has relatively high response (36%) and disease (81%) control rates but is generally not curative and many patients will experience recurrent disease and/or metastasis. Therefore, there is a great need to identify predictive biomarkers for recurrence and disease progression in cetuximab-treated R/M HNSCC patients to facilitate patient management and allow for treatment modification. In R/M HNSCC patients treated with cetuximab-based chemotherapy including the EXTREME regimen and cetuximab+platinum+paclitaxel/pemetrexed, we show increased gene expression of IL-1 ligands in baseline tumor samples in patients with long PFS (> 12 months) compared to short PFS (< 5.6 months) (Fig. 3). Note that in the EXTREME trial, the median PFS obtained with the EXTREME regimen was 5.6 months [51]. Therefore increased genetic expression of IL-1 ligands may predict a favorable PFS in patients treated with this regimen that is above and beyond the median PFS reported in the EXTREME trial. These results are supported in a separate cohort of R/M HNSCC patients that were treated with similar regimens described in Fig. 4. In this case we show that high serum levels of activating IL-1 ligands were associated with favorable PFS compared to low (Fig. 4c,d). These data support the tumor gene expression results observed in Fig. 3 which is promising since a serum biomarker would be ideal, as blood tests are relatively non-invasive and inexpensive compared to genetic analyses from tumor biopsies.

As mentioned before, PFS in the EXTREME trial was quite short (median survival = 5.6 months) and cetuximab-refractory R/M HNSCC patients have few remaining therapy options. Therefore a number of clinical studies have been carried out with the addition of experimental agents to cetuximab therapy with the purpose of overcoming resistance to cetuximab and increasing survival. In a cetuximab-refractory R/M HNSCC patient cohort treated with cetuximab and ficlatuzumab, we showed that high baseline serum levels of IL-1 ligands were associated with a more favorable PFS compared to low serum levels (Fig. 5c,d). Although the differences in PFS were not significant according to the conventional log rank test used in assessing survival outcome differences, we did observe a late separation of the curves (Fig. 5c,d) which is promising and typical of cancer clinical trials utilizing immunotherapy agents that are considered successful [52]. These results may indicate even in cetuximab-refractory patients that IL-1 ligands may still be associated with favorable/durable clinical outcomes in a subset of patients treated with cetuximab-based therapies.

It is already well known that NK cell activity is a major mechanism of action of cetuximab (in addition to EGFR inhibition). The ability of IL-1 to predict favorable survival outcomes to cetuximab-based therapy may be due to the role of IL-1 in mediating NK cell activity triggered by cetuximab. It is possible that IL-1 signaling is necessary for optimal cetuximab-induced NK cell activity and anti-tumor efficacy which is supported by our data showing that anakinra both suppresses number of activated NK cells induced by cetuximab (Fig. 6) and partially (but significantly) reverses the anti-tumor efficacy of cetuximab in athymic nude mice (Fig. 7) where NK cells are present. These results represent a potential explanation of why low gene or circulating IL-1 levels may be associated with poor survival outcomes to cetuximab-based therapy.

Altogether, we recognize that sample size is a major limitation in all of these data sets analyzed in these studies and our intention is not to draw concrete conclusions from this work. Our goal is to provide preliminary evidence from several different studies related to the promising nature of IL-1 ligands as predictive biomarkers of cetuximab response. Collectively, our results suggest that IL-1 ligands are worthy of further investigation as predictive biomarkers of cetuximab response and our future goals are to validate these findings in larger clinical trials.

## Conclusions

Here we provide preliminary evidence that tumor gene expression and/or circulating levels of activating IL-1 ligands may be associated with HNSCC tumor response to cetuximab. While our results were obtained from small datasets, the consistent and promising nature of our findings with IL-1 ligand expression and response to the EXTREME regimen and other cetuximab-based chemotherapy warrants further investigation into its potential use as a biomarker in a larger cohort of HNSCC patients treated with cetuximab-based therapy. Beyond HNSCC, this work may lay a foundation to study IL-1 ligands as biomarkers for responsiveness to cetuximab in colorectal cancer and in immune activating agents in other disease sites as well. Overall, these results support the further investigation of IL-1 ligands as predictive biomarkers for cetuximab responsiveness in HNSCC.

## Data Availability

The datasets used and/or analyzed during the current study are available from the corresponding author on reasonable request.

## Abbreviations

^18^FDG-PET: 2-deoxy-2-(^18^F)fluoro-D-glucose- positron emission tomography
ADCC: Antibody-dependent, cell-mediated cytotoxicity
CRC: Colorectal cancer
EGFR: Epidermal growth factor receptor
EXTREME: ERBITUX in first-line Treatment of REcurrent or MEtastatic head and neck cancer
FDR: False discovery rate
HNSCC: Head and neck squamous cell carcinoma
IL-1: Interleukin-1
IL-1R1: Interleukin-1 receptor type 1
IL-1RA: Interleukin-1 receptor antagonist
IL-1α: Interleukin-1 alpha
IL-1β: Interleukin-1 beta
NSCLC: Non-small cell lung cancer
PD: Progressive disease
PFS: Progression-free survival
PR: Partial response
R/M: Recurrent and metastatic
SD: Stable disease
SUV: Standard uptake value
TCGA: The Cancer Genome Atlas
